# PEM Anchorage on Titanium Using Catechol Grafting

**DOI:** 10.1371/journal.pone.0050326

**Published:** 2012-11-30

**Authors:** Hélène Marie, Amélie Barrere, Frédérique Schoentstein, Marie-Hélène Chavanne, Brigitte Grosgogeat, Laurence Mora

**Affiliations:** 1 Université Paris 13, Sorbonne Paris Cité, BPC, Villetaneuse, France; 2 Univ Paris Diderot, Hemostasis Bio-engineering and Cardiovascular Remodelling, Paris, France; INSERM, U698, Paris, France; 3 Université Paris 13, Sorbonne Paris Cité, Laboratoire des Sciences des Procédés et des Matériaux, CNRS (UPR 3407), Villetaneuse, France; 4 UMR CNRS 5615 Laboratoire des Multimatériaux et des Interfaces, UFR d’Odontologie, Université Lyon 1, Lyon SCTD, Hospices Civils de Lyon, Lyon, France; Université de Technologie de Compiègne, France

## Abstract

**Background:**

This study deals with the anchorage of polyelectrolyte films onto titanium surfaces via a cathecol-based linker for biomedical applications.

**Methodology:**

The following study uses a molecule functionalized with a catechol and a carboxylic acid: 3-(3,4-dihydroxyphenyl)propanoic acid. This molecule is anchored to the TiO_2_ substrate via the catechol while the carboxylic acid reacts with polymers bearing amine groups. By providing a film anchorage of chemisorption type, it makes possible to deposit polyelectrolytes on the surface of titanium.

**Principal Findings:**

Infrared spectroscopy (ATR-FTIR), X-ray photoelectron spectroscopy (XPS), contact angle and atomic force microscopy (AFM) measurements show that the different steps of grafting have been successfully performed.

**Conclusions:**

This method based on catechol anchorage of polyelectrolytes open a window towards large possibilities of clinical applications.

## Introduction

Biomaterials are natural or synthetic materials for medical use. They are used to replace, supplement or cure any tissue, organ or function of a living organism, in order to work in close contact with living tissues or biological fluids. Their use for therapeutic purposes is not recent, but their development and efficiency are new (medical advances, industrial research). Many biological, chemical and physical criteria have to be taken into account in the design of a relevant biomaterial.

Titanium is a biomaterial commonly used in clinical applications (orthopedic, dental and cardiovascular implants) whose biocompatibility and corrosion resistance are admitted [1]. However, the integration of this metal in human body is not yet optimal. Interfaces properties being fundamental for biomaterials [2], improvement of titanium surface elaboration is a strong line of research in development.

Covering titanium surfaces by one or more layers of polymers creates new outlook for titanium-biological environment interfaces. Thus, various studies have examined the use of polyelectrolytes films for materials covering [3]–[5]. According to vascular or bone system applications, the film properties are expected to be different. The technique commonly used to coat a substrate with polyelectrolytes is based on electrostatic interactions. The strength of these interactions against the stress applied to the biomaterial may be insufficient [6]. Therefore, in order to anchor onto the titanium surface a film of synthetic or natural polyelectrolytes, a new technique must be implemented.

Thus, the grafting technique is fundamental especially to guaranty a sufficient chain surface density. Different types of grafting methods are available. One way to perform grafting on a titanium surface is to use silanes [7]. With this technique, a silane is first grafted to the surface of the substrate and reacts in a second step with a polymer.

Another way is grafting using catechols. The last ten years have seen a trend of grafting oriented towards biomimetic surfaces. Thus, Dalsin et al [8] were inspired by mussel adhesive proteins. These shells are indeed known to adhere to various surfaces such as rocks, wood, boat hulls polymer … Mussels secrete a fluid rich in adhesive protein that solidifies quickly and has remarkable cohesive and adhesive properties. These properties were related to the presence of an amino acid: dopamine (L-3,4-dihydroxyphenylalanine). Although the mechanism of adhesion of mussels is not yet understood, the scientific community speculates on chemical interactions between catechol function of dopamine and the various surfaces. Thus, functionalizing a polymer with a catechol group would anchor it on many surfaces [9].

Several studies have focused on the study of interactions between catechol and surface of TiO_2_. The reaction is still not fully explained. Based on the molecular orbital theory and on the theory of functional density, Redfern and al [10] modeled the interaction between catechol and TiO_2_ nanoparticles. It follows from calculations carried out, that catechol reacts easily with Ti = O surface sites to form a bidentate structure where two atoms of the catechol cycle are in connection with two titanium atoms belonging to the titanium surface. These results are, however, tarnished by the number of approximations made and the lack of experimental evidence. According to Persson et al [11], there is a strong electronic coupling between catechol and TiO_2_ and the interaction between them is a strong chemisorption. The structure studied is, by default, bidentate bridging. The energy calculations corroborate the thesis of an electronic transfer that is different than for usual models (direct transfer of the molecular orbital HOMO of catechol in the band conduction of TiO_2_) and agree to the absorbance measurements. Therefore, a track of investigation is opened on the catechol geometry of adsorption on the surface of TiO_2_. Results observed in UV photoelectronic spectroscopy can only be explained by the simultaneous existence of two structures: monodentate and bidentate. Catechols are distributed as a monolayer on the surface according to these two structures, both inclined 15° to 30° from the normal surface [12].

This technique of surface anchoring is increasingly used. A distinction is commonly made between three different approaches. The first one is the direct polymerization from the substrate surface by using an initiator bringing a catechol group. Another approach is to functionalize a polymer with a molecule loading a catechol group and then to anchor the polymer onto the surface of a material [13], [14]. A third type of approach is suggested by Chua et al [15]. Dopamine is first anchored to the surface of TiO_2_, then, by reaction with glutaraldehyde an aldehyde functionalized surface is obtained, able to react with amine containing polymers.

Inspired by this work, our study is based on a bifunctional molecule: 3-(3,4-dihydroxyphenyl)propanoic acid, used as a linker between TiO_2_ surface and polyamine chains, thanks respectively to its catechol and to its carboxylic acid. In this way, the additional step involving glutaraldehyde, known for its toxicity, is suppressed. Polyelectrolytes films were built from this surface and were analyzed.

## Materials and Methods

### Preparation of Titanium Samples, Grafting and Films Polyelectrolytes

#### Preparation of samples of titanium

Samples are pure titanium (TiCP provided by Timet Savoie, France). These are discs of 10 mm diameter and 2.7 mm thickness. Before use, samples were manually polished with abrasive discs P1000 to P4000 (Struers, France). After use and in order to be recycled, the samples were polished again and undergo the same protocol as described above. Cleaning-degreasing is applied to pellets in several ultrasonic baths to eliminate surface impurities: fifteen minutes with acetone and then rinsing with distilled water and dry N_2_ gas; fifteen minutes with ethanol and then rinsing with distilled water and drying N_2_ gas, fifteen minutes in distilled water and then a rinsing with distilled water and drying N_2_ gas. The samples thus prepared will be marked TiO_2_.

**Figure 1:**
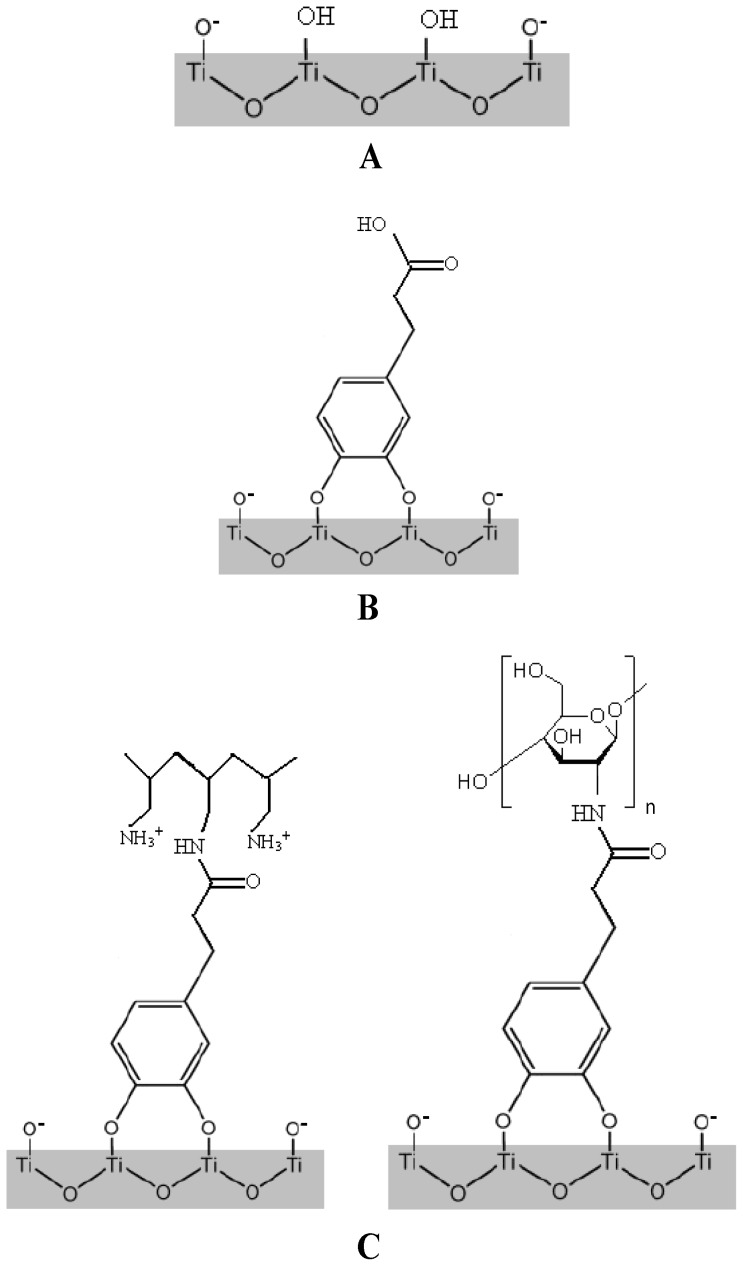
Grafting of catechol groups onto titanium surface. (A) Initial Titanium surface exhibits OH groups that will be used to interact chemically with catechol cycle: TiO_2_ (B) Catechol is bounded to titanium surface and exhibits the carboxylic group COOH: TiCOOH (C) Poly(allylamine hydrochloride) (PAH) and Chitosan are finally covalently bounded to catechol. The COOH group has been used to react with the polycation and its amine group. The two polycations presented are polyallamine hydrochloride (PAH) and Chitosan, left and right respectively: (Left) TiCOOPAH; (Rigth) TiCOOchit.

#### Preparation and grafting of catechol

The catechol group is localized on the acid 3-(3,4-dihydroxyphenyl) propanoic acid (Alfa Aesar): the DHPP. The DHPP is dissolved in distilled water (1 mg/mL). The titanium sample is deposed on a Teflon specimen holder, so that the sample is coated on both sides, and placed in this solution, overnight in the dark. The sample is then rinsed with distilled water, N_2_ gas dried, washed in distilled water fifteen minutes in an ultrasonic bath, rinsed again with distilled water and finally dried with N_2_ gas. Titanium that has reacted with the DHPP will be noted as to TiCOOH.

**Figure 2:**
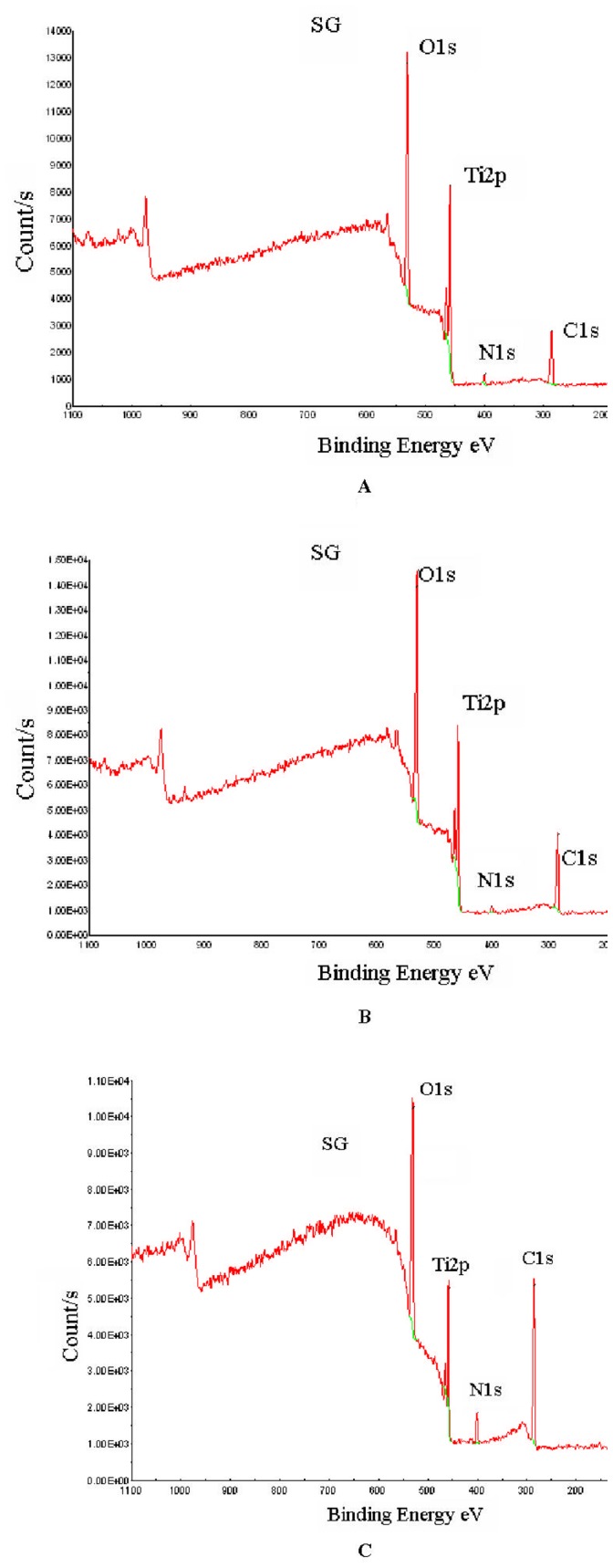
XPS spectra for titanium surface analysis. (A) initial surface: TiO_2_, (B) Titanium with catechol grafted at its surface: TiCOOH, (C) Titanium with catechol bonded to PAH: TiCOOPAH.

**Figure 3:**
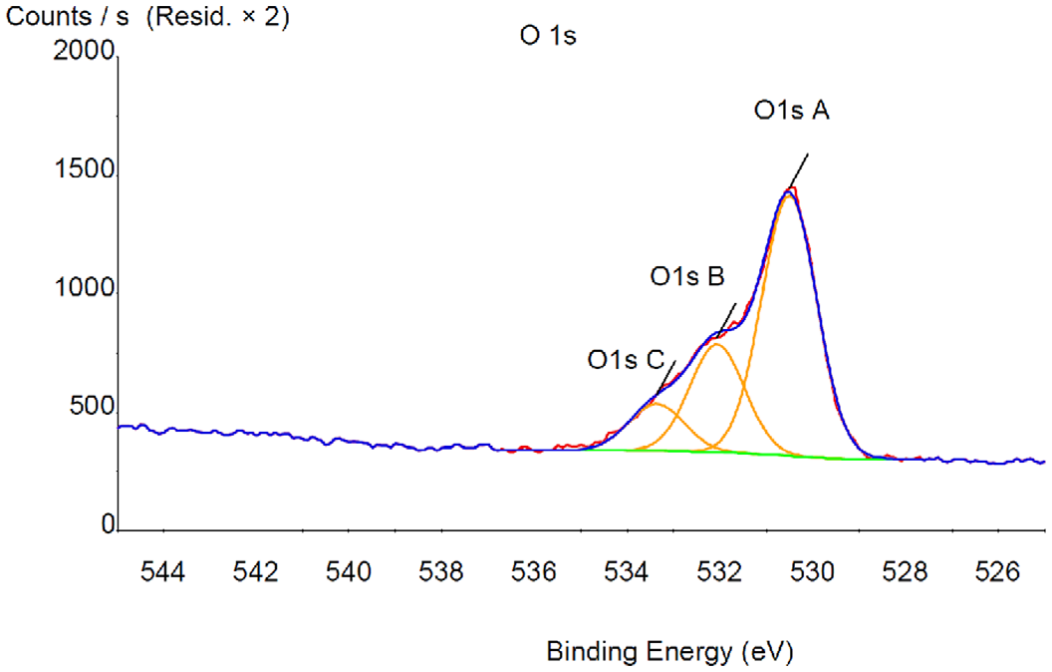
X-Ray Photoelectron Spectroscopy (XPS) modelisation for Titanium with catechol (TiCOOH) in order to extract the atomic % of each element presented in **table 1**.

The sample of titanium coated with DHPP and the polyallylamine, figure 2C, shows an increase in the amount of carbon as well as that of nitrogen (respectively 53.7% and 5.2% in table 1). These peaks result from amine and amide groups as well from polymer skeletal hydrocarbons. The decrease in the intensity of the titanium combined with the significant increase in nitrogen and carbon C-C clearly show the presence of nitrogen-containing polymer on the surface, confirming thus the fixation of PAH onto TiCOOH (Fig. 6).

#### Preparation of solutions and polyelectrolyte multilayer films

Two types of polycation are used: poly (allylamine hydrochloride) (PAH, Mw = 15 kDa, Aldrich) and chitosan (chit, Mw = 400 kDa, Sigma, France). The polyanion is poly (sodium 4-styrene sulfonate) (PSS, Mw = 70 kDa, Aldrich). The polyelectrolyte concentrations are 5 mg/mL.

**Figure 4:**
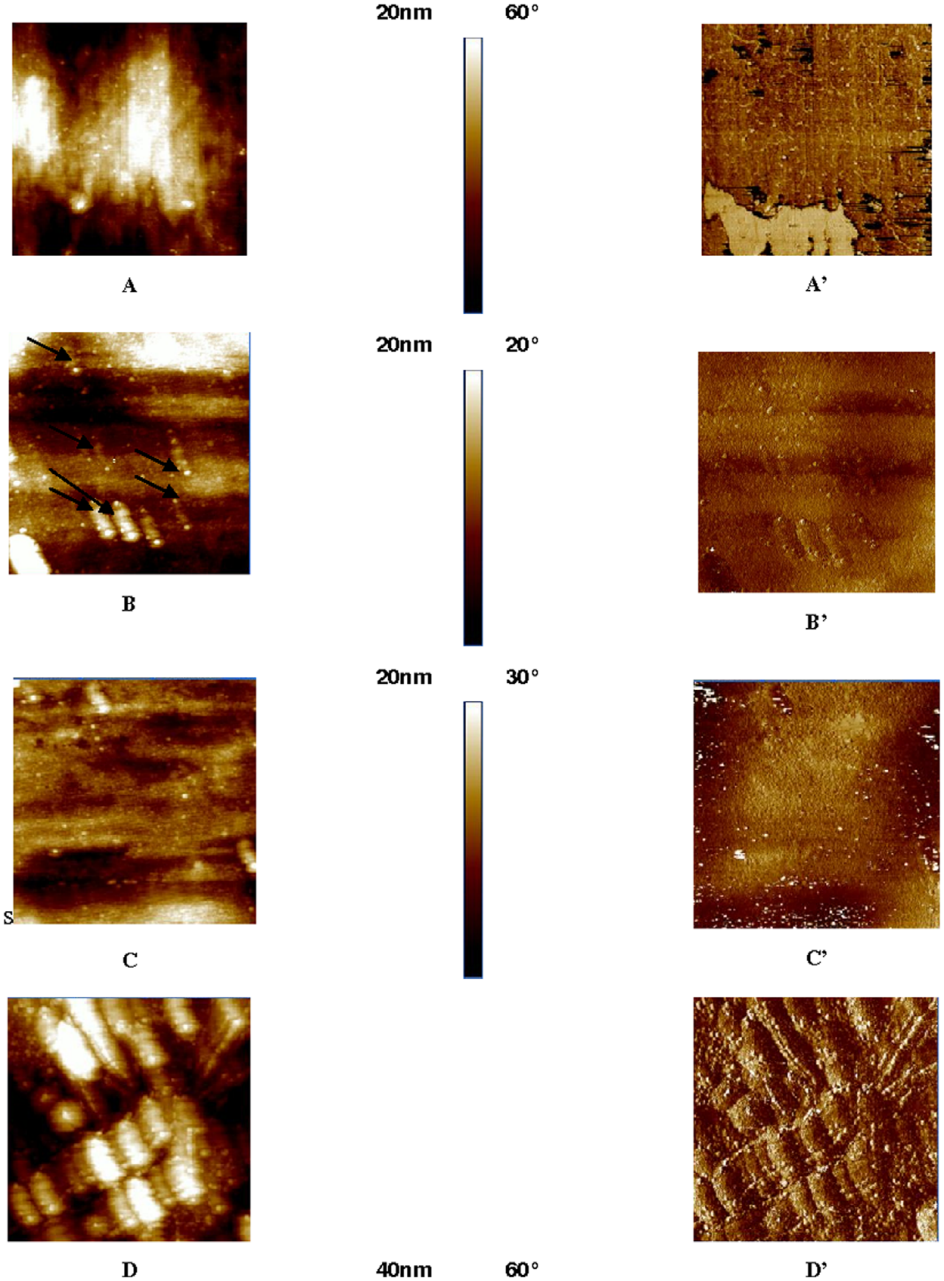
AFM images acquired in tapping mode of titanium surface, titanium with catechol anchored, titanium with catechol covalently bonded to PAH, and finally with PEM physisorbed (PAH-PSS)_7_. A, A′: TiO_2_. B, B′: TiCOOH. C, C′: TiCOOPAH. D, D′: TiCOO(PAH-PSS)_7_. For X = A, B, C or D: X = 2D topographic image, X′ = phase.

After grafting of DHPP on titanium surface, a first layer of polycation is incubated in the presence of N-(3-dimethylaminopropyl)-N′- ethylecarbodiimide (EDC, Acros Organics), 125 mg/mL, in a solution of polycation at 5 mg/mL. EDC is used to facilitate the covalent bonding between the catechol carboxylic groups of and the polycation amine groups. The sample is placed on the Teflon holder in this solution for 2 hours, with a slow stirring. The sample is then rinsed with distilled water, dried with N_2_ gas, washed in distilled water fifteen minutes in an ultrasonic bath, rinsed again with distilled water and finally dried with N_2_ gas. After reaction with PAH or chitosan, the samples will be noted TiCOOPAH and TiCOOchit respectively.

The formation of subsequent layers of polyanion/polycation is obtained by successive adsorption of each polyelectrolyte. The sample is placed in the solution for twenty minutes and then rinsed with distilled water for two minutes and dried in N_2_ gas. The final architecture are noted: TiCOO(PAH/PSS)_n_ and TiCOO(chit/PSS)_n_ where n is the number of layers.

**Figure 5:**
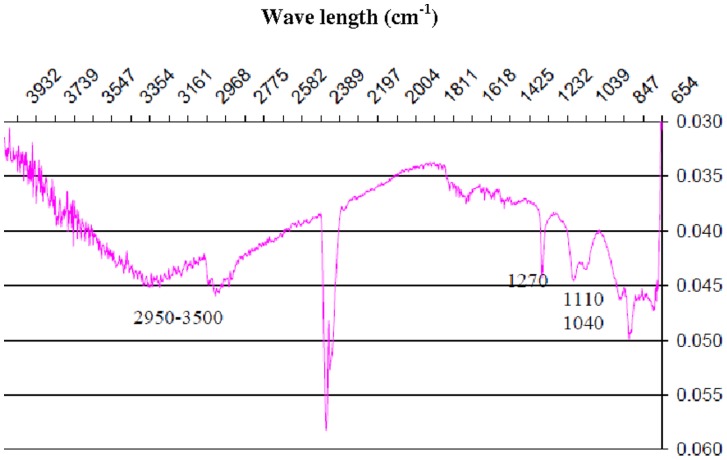
ATR-FTIR, TiCOOchit Spectra.

**Figure 6:**
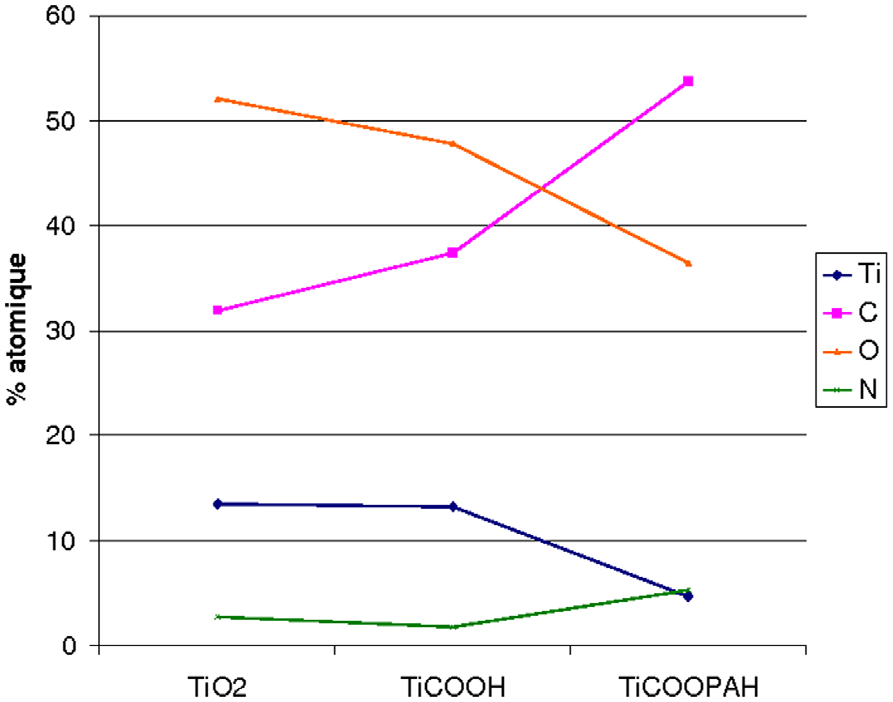
Graphic. Atomic % for the different surfaces.

### Surface Characterizations

#### Infrared spectroscopy (ATR-FTIR) analysis of the polyelectrolyte films

This technique allows us to analyze the chemical functions exhibited on the surface via vibration characteristics of chemical bonds. The analysis is performed by the FTIR spectrometer (Nicolet, Avatar 370). The spectra are carried out at a resolution of 4 cm^−1^ on a Ge crystal between 4000 cm^−1^ and 600 cm^−1^.

#### X-ray photoelectron spectroscopy (XPS)

The analysis of the sample surface chemical composition was obtained by XPS Spectrometer (VG 2201), under ultrahigh vacuum, using a monochromatic source of Al Kα 1486.6 eV. The C1s peak (CC bond) is used as reference (284.8 eV).

#### Contact angle measurements

Measurements of static contact angle are made by the sessile drop method. The measurement system is a DSA10, Krüss GmbH. A drop of 2 µL of distilled water is deposited on the sample surface through a syringe. The image of the drop is captured and the contact angle measured using the software “Drop Shape Analysis.” Before measurement, samples were rinsed in distilled water and dried under N_2_. Contact angles are measured in air at 21°C. Ten contact angles measurements were performed for each condition (n = 10).

#### Analysis of the polyelectrolytes films morphology and roughness by atomic force microscopy (AFM)

AFM was used to perform 2D topographic images of samples. The AFM was a nanoscope 5 from Bruker-Nano and the cantilever was a silicon probe with aluminium reflex coating (resonant frequency: 300 KHz). The constant force of the cantilever was 40 N/m.

Images were made in air at room temperatures and in tapping mode. Surface feature size and roughness parameters were therefore determined by the AFM software program. The images of topography and deflection were carried out simultaneously with a resolution of 256 * 256 pixels. Roughness surface was calculated from 10 µm×10 µm images.

### Statistical Analysis

The statistics used here for contact angle measurements was based on a comparison of variances and means of two populations by the Student test (t-test with Excel software), n = 4. The probability of correlation is based on the Pearson coefficient (p), which value, when less than 0.01, corresponds to a statistically significant difference between the two populations of values that are compared.

**Table 1:** XPS quantification for (A) TiO_2_, (B) TiCOOH, (C) TiCOOPAH (D) in atomic %.

	Ti			
Atomes	Ti	O	C	N
**atomique%**	13.43	52.03	31.87	2.67
	**TiCOOH**			
**Atomes**	**Ti**	**O**	**C**	**N**
**atomique%**	13.2	47.73	37.34	1.72
	**TiCOOPAH**			
**Atomes**	**Ti**	**O**	**C**	**N**
**atomique%**	4.65	36.4	53.72	5.22

## Results

To anchor a polycation such as PAH or a natural polymer such as chitosan, onto the surface of TiO_2_ and using biomimetic catechols anchorage, two solutions can be considered: either a functionalization of the polymer (“grafting onto” strategy) or an anchorage onto a functionalized substrate (“grafting from” strategy).

According to the “grafting onto” strategy, catechol functionalized polymers can be chemisorbed on a TiO_2_ surface (figure 1A and 1B). In this way, the DHPP can be condensed on a polymer bearing amine groups in presence of a catalytic system (Figure 1C). To facilitate the reaction, poly(allylamine hydrochloride) was chosen as polycation for its high concentration of amines, which can potentially lead to a higher density of anchoring. However, given the difficulties of precipitation, the amines degradation and the incomplete catalysts purification of the polymer, this way has no longer been explored and “grafting from” strategy was developed.

This second approach consists first in the anchoring of DHPP onto TiO_2_ surface by simple dipping of the discs in a DHPP solution, followed by the condensation of PAH on the carboxylic acids present at the discs surface. The final polyelectrolytes multilayer is obtained after successive dippings in poly(sodium 4-styrene sulfonate) and PAH solutions. Several methods were used at each step of the surface modification to characterize the coating of TiO_2_.

### Contact Angle Measurements

Once the DHPP grafted, the surface exhibited a contact angle of 51.3±2° whereas its value was of 57.0±4° for TiO_2_ and 70.4±5.7° after reaction with the polyallylamine.

### XPS Analysis

XPS surface analysis was performed on TiO_2_, TiCOOH and TiCOOPAH surfaces (figure 2). The uncoated titanium sample, Figure 2A shows the presence of four elements. The binding energies are 284.8 eV (carbon), 458.6 eV (titanium), 530.2 eV (oxygen), 399.9 eV (nitrogen). Titanium and oxygen are due to the oxide layer covering the titanium. Carbon comes from an inevitable hydrocarbon contamination due to adsorption from the ambient air and accidental contamination (rinses with various solvents, sample transport etc.). Nitrogen is also present in small amount. The titanium sample reacted with the DHPP is showed in Figure 2B and presents the same elements. In order to verify that DHPP is well fixed onto titanium surface, XPS spectra modeling was performed. The results effectively showed a peak at 533.4 eV corresponding to the carboxylic acid (Figure 3).

### AFM Images

AFM images presented in figure 4 show a rough sample of TiO_2_ (surface roughness of 35 nm). Subsequently, the images of surface covered with TiCOOPAH indicate a multitude of buds on the surface, attributed to a successful deposit of PAH (Ra = 15 nm). The evolution of the titanium surface after the various deposits is observable through 2D images (Figure 4 A-D to Figure 4 A′–D′). Figure 4 A, A′ shows the titanium surface of reference: the surface is homogeneous without defects, a few polishing scratches can still be distinguished. Figure 4 B, B′ is the titanium surface after reaction with the DHPP, small nodules (diameter of the order of nm) appearing, showing a new surface morphology. The surface roughness is 11.9 nm. AFM images show that the recovery by the DHPP is uniform. Subsequently, the condensation of PAH on the carboxylic acid shows the DHPP nodules and the apparent roughness of 12 nm (Figure 4 C, C′). This same sample coated with (PAH-PSS)_7_ (Figure 4 D, D′) still has small nodules which were added to larger structures (size of the order of hundreds of nm). Roughness increased to 23 nm.

### **ATR-FTIR Analysis**

As the Angulator of the device does not, in the majority of cases, permit the analysis of thin films, FTIR have been only used to characterize chitosan based coating. Indeed, TiCOOchit (figure 5) was sufficiently thick to be detected by ATR-FTIR. Except for absorption at 2345 cm^−1^ due to CO_2_, the spectrum shows a broad band between low intensity 2950 cm^−1^ and 3500 cm^−1^ that can be attributed to vibrations for N-H and O-H bonds respectively. Moreover, several thin absorption bands at 1270 cm^−1^, 1110 cm^−1^ and 1040 cm^−1^, are attributable to stretching vibrations of C-O bonds, to the deformation of O-H bonds in the plan for primary and secondary alcohols [16], [17].

## Discussion

### Titanium Biomaterials

Titanium has attractive mechanical properties and low reactivity with human tissues. It is highly resistant to corrosion and has a low density (4,51 g.cm^−3^). These properties facilitate its use in the biomedical field. In addition, the surface of Titanium is covered with an oxide layer promoting integration and lifetime of the metal in the human body. Whatever it is, a biomaterial is a foreign body and its introduction into the body causes a more or less reaction of the environment. Thus, the biocompatibility evaluates the compatibility of a natural or synthetic material and a biological system in short, medium and long term. The aim is to observe the effects of the implant on the living (proteins or cells) and conversely the effects of living on the implant. In general, the biocompatibility is modulated by the biomaterial surface (interfacial) or structural properties. Although biocompatible, titanium exhibits a thrombogenicity that can generate the formation of a blood clot [18]. The modification of its surface can modulate not only its integration into the body but also bacterial adhesion [19], [20], or even make it bioactive. The surface properties of titanium used in biomedical applications can be improved by its covering by a (macro) molecules layer. Thus, much research has turned to the use of polyelectrolyte films.

### Polyelectrolytes Multilayers (PEM)

The deposition of polyelectrolyte using layer by layer (LBL) technique was described in 1997 by Decher [21], . The assembly is made by successive dipping the sample alternatively into a solution of polyanion and polycation**.** These dips are separated by rinsing steps. Growth of these films, layer by layer, is based on the charge excess that appears after each layer of polyelectrolyte of opposite sign. The occurrence of the charge excess was demonstrated by the alternation of the surface zeta potential during the construction of such films [23], [24]. As to the construction of the polyelectrolyte film, its mass and thickness increase. The construction of a polyelectrolyte multilayer is now a well-established concept for biomaterials covering. In addition to this easy film elaboration, polyelectrolytes offer a wide variety of molecules. Thus, using such film can improve cell adhesion and/or, if necessary, reduces bacteria adhesion.

Polymers are extensively used to construct polyelectrolytes films and they must essentially offer a large surface density of chain on the substrate. The existence of electrostatic forces, to anchor a film of polyelectrolyte is often appropriate. For example, the titanium surface beeing negatively charged, the first polycation deposited is adsorbed due to electrostatic interactions. The most current polycation used in this case is poly(ethyleneimin). However, with such interactions, films deteriorate gradually and tend to disappear [25], [26].

PAH containing numerous amine groups is mainly used in this study and will be associated to PSS witch is often coupled to PAH in literature. These two polyelectrolytes are widely used in biomaterial research due to their good biocompatibility exhibiting for example a high proliferation and adhesion compromise for dental fibroblast cells [27].

Chitosan is a natural polycation with very interesting properties for medical applications. This polymer has the ability to improve cell proliferation of dental cells [28], [29]. Moreover, its positive charge, due to amine groups, allows its coupling with PSS and also induces antibacterial characteristics. This biopolymer is thus well adapted for implant coatings applications [30].

In this paper, the ability of the grafting was mainly assessed with PAH/PSS based films. However, Chitosan, due to its interesting properties was also studied and should confirm the results obtained with PAH/PSS. Indeed, the chitosan ATR-FTIR spectrum confirms therefore the deposition of chitosan on the sample. This could verify its anchorage.

#### Direct polymerization on the substrate surface

This approach requires the use of a molecule bringing a catechol group and a polymerization initiator group. This molecule is initially bonded to titanium. Then, polymerization is initiated directly from titanium [31]. This technique is simple to implement and can be applied to many inorganic surfaces with several types of monomers and solvents.

#### Polymer functionalization

Another approach is to functionalize a polymer with a molecule loading a catechol group and then to anchor the polymer to a surface of a material [9], [13], [14]. For example, Saxer et al. [13] proposed an original approach combining electrostatic adhesion of a polyelectrolyte and anchorage of a catechol function on titanium. Thus, the (3,4-dihydroxyphenyl) acetic acid, carrier of a catechol function, reacts with polymer, poly (L-lysine)-poly (ethylene oxide) to form an amide by condensing the amine function of the polymer and its carboxylic acid.

The polymer is then contacted with the substrate, which is sufficient to anchor the polycation: chemisorptions’ anchoring through the catechol type and electrostatic interactions between the polycation and the negatively charged surface of titanium. Functionalization of the polymer by a catechol creates a new class of polymers capable of forming a polyelectrolyte film while ensuring a stable and strong adhesion to the substrate.

#### Anchorage onto functionalized substrate: grafting “onto”

A third type of approach is suggested by Chua et al [15]: a catechol group is anchored to the surface of TiO_2_ and then reacted with a polycation. Chua and al used glutaraldehyde to form an amide (between the functionalized catechol molecule, -NH_2_ and NH_2_-functionalized polycation) to graft the polymer on the surface of the sample.

Scanning tunneling microscopy measurements show that molecules of catechol form a dense monolayer on the TiO_2_ surface.

The decrease in roughness after reaction with the DHPP confirms the setting of the molecule on the substrate. On the other hand, the nodules visible on the surface after addition of DHPP but also after reaction with PAH, as well as TiCOO (PAH-PSS)_7_ are similar to those already observed for a film with PAH and PSS [32].

For wettability measurements, once the DHPP grafted, the surface was substantially more hydrophilic (θ = 51.3±2°) than TiO_2_ (57.0±4°). Conversely, after reaction with the polyallylamine, the surface is more hydrophobic (θ = 70.4±5.7°): the hydrocarbon skeleton of the polymer makes the surface hydrophobic. The average contact angles measured between TiCOOH and TiCOOPAH are, according to a Student’s t test, significantly different. Thus, the surface has different wetting properties, which confirms the presence of polyallylamine.

The evolution of the surface morphology of the different steps of titanium surface grafting observed by AFM confirms the final PEM anchorage. As Cai et al [4] have postulated, changes in topography (here the formation of ellipsoids) could have origin in the difference between the molecular weight of PAH (MW = 15 kDa) and PSS (Mw = 70 kDa).

This approach commonly known as “grafting onto” allows a grafting density higher than for the previous approach. However, the glutaraldehyde is highly toxic and is not recommended for use with biomedical materials. The potential biomedical applications are numerous and concern all domains where implants have to be covered by a bioactive layer. But the real improvement that has to be pointed out is in the applications where a strong mechanical connection to the bare biomaterial of the functionnalization layer is particularly expected such as in cardiovascular, orthopedic or dental domains (internal and/or external implantation). Indeed, blood flux in vessels or tribological stresses in bones connected to the bioactive implants, increase drastically the necessity to anchorage strongly any bioactive coating to the biomaterials. Moreover, in the case of PEM films, a bulk reticulation of the successive layers of the film can be performed to enlarge the mechanical resistance to scratch to the overall coating [33].

Concerning biocompatibility of the final coating, PEM will totally recover the catechol groups because of the thickness of conventional PEM films (containing at least 4 bi-layers to ensure a steady state in chemical composition [34]). The nature of the PEM film that is conditioned by the specific application of interest will modulate cell response to the coating. In this study no particular application was selected and PEM can be applied in a large range of biomedical domain. If a biodegradable PEM was involved in the design biomaterial, cell response should then be tested in term of biocompatibility.

### Conclusion

This study was devoted to anchor a first polyelectrolyte layer on the surface of TiO_2_. For this, the biomimetic approach was preferred. The anchorage of a molecule having a catechol group followed by the condensation of a polycation on this molecule ensures a strong interaction between the substrate and the first layer of polyelectrolyte. The anchored layer onto titanium has resisted to washes with ultrasound which have been imposed. Subsequently, XPS analysis has confirmed the successive deposits of DHPP and PAH, in agreement with the results of wettability. The successive deposits were observed in AFM, on the same sample. It appeared that, with the experimental conditions established, the DHPP covers uniformly the surface of TiO_2_. Measurements of contact angle and AFM showed that wettability and roughness properties of the surface varied with the recovery. On the other hand, the successive deposition of polyelectrolytes is causing an evolution of surface morphology: the ellipsoid-shaped structures appear, perhaps due to different chain lengths of polyelectrolyte’s used.

The objective of this study was to provide a substrate binding of the first PEM layer stronger than for a simple classical physisorption, thanks to catechol groupments. This open possibility in numerous domains of clinical applications due to the large variety of PEM nature available (from synthetic to natural or biodegradable ones).

This line of research is fairly recent; it seems useful that the next steps are devoted to screening the many parameters involved in. Thus, concentrations of DHPP and polyelectrolytes should be varied and the morphologies of the obtained surfaces studied. This would allow for example to study the evolution of the size of the nodules observed by AFM in this study. The reaction time should be optimized according to densities of grafting and film thicknesses desired. Finally, the initial roughness of titanium may also play a role for a physical anchor and it would be interesting to see if the combination of a physical and the chemical anchoring is feasible and in what extent it is interesting to fight the possible desorption of the film.
