# Correction: PEM Anchorage on Titanium Using Catechol Grafting

**DOI:** 10.1371/annotation/74fc701c-b7e5-445e-aa37-34bed37a3801

**Published:** 2013-06-07

**Authors:** Hélène Marie, Amélie Barrere, Frederic Schoenstein, Marie-Hélène Chavanne, Brigitte Grosgogeat, Laurence Mora

The third author's name was spelled incorrectly. The correct name is Frederic Schoenstein. The correct citation is Marie H, Barrere A, Schoenstein F, Chavanne M-H, Grosgogeat B, et al. (2012) PEM Anchorage on Titanium Using Catechol Grafting. PLoS ONE 7(11): e50326. doi:10.1371/journal.pone.0050326 

